# Redirecting pantoprazole as a metallo-beta-lactamase inhibitor in carbapenem-resistant *Klebsiella pneumoniae*

**DOI:** 10.3389/fphar.2024.1366459

**Published:** 2024-03-12

**Authors:** Wesam H. Abdulaal, Nabil A. Alhakamy, Amer H. Asseri, Mohamed F. Radwan, Tarek S. Ibrahim, Solomon Z. Okbazghi, Hisham A. Abbas, Basem Mansour, Aly A. Shoun, Wael A. H. Hegazy, Mahmoud Saad Abdel-Halim

**Affiliations:** ^1^ Department of Biochemistry, Faculty of Science, Cancer and Mutagenesis Unit, King Fahd Medical Research Center, King Abdulaziz University, Jeddah, Saudi Arabia; ^2^ Department of Pharmaceutics, Faculty of Pharmacy, King Abdulaziz University, Jeddah, Saudi Arabia; ^3^ Center of Excellence for Drug Research and Pharmaceutical Industries, King Abdulaziz University, Jeddah, Saudi Arabia; ^4^ Mohamed Saeed Tamer Chair for Pharmaceutical Industries, King Abdulaziz University, Jeddah, Saudi Arabia; ^5^ Biochemistry Department, Faculty of Science, King Abdulaziz University, Jeddah, Saudi Arabia; ^6^ Centre for Artificial Intelligence in Precision Medicines, King Abdulaziz University, Jeddah, Saudi Arabia; ^7^ Department of Pharmaceutical Chemistry, Faculty of Pharmacy, King Abdulaziz University, Jeddah, Saudi Arabia; ^8^ Global Analytical and Pharmaceutical Development, Alexion Pharmaceuticals, New Haven, CT, United States; ^9^ Microbiology and Immunology Department, Faculty of Pharmacy, Zagazig University, Zagazig, Egypt; ^10^ Pharmaceutical Chemistry Department, Faculty of Pharmacy, Delta University for Science and Technology, Gamasa, Egypt; ^11^ Microbiology and Immunology Department, Faculty of Pharmacy, El Salehey El Gadida University, Sharkiya, Egypt; ^12^ Pharmacy Program, Department of Pharmaceutical Sciences, Oman College of Health Sciences, Muscat, Oman

**Keywords:** *Klebsiella pneumoniae*, pantoprazole, carbapenems, metallo-β-lactamase inhibitor, healthcare

## Abstract

The development of resistance to carbapenems in *Klebsiella pneumoniae* due to the production of metallo-β-lactamases (MBLs) is a critical public health problem because carbapenems are the last-resort drugs used for treating severe infections of extended-spectrum β-lactamases (ESBLs) producing *K. pneumoniae*. Restoring the activity of carbapenems by the inhibition of metallo-β-lactamases is a valuable approach to combat carbapenem resistance. In this study, two well-characterized clinical multidrug and carbapenem-resistant *K. pneumoniae* isolates were used. The sub-inhibitory concentrations of pantoprazole and the well-reported metallo-β-lactamase inhibitor captopril inhibited the hydrolytic activities of metallo-β-lactamases, with pantoprazole having more inhibiting activities. Both drugs, when used in combination with meropenem, exhibited synergistic activities. Pantoprazole could also downregulate the expression of the metallo-β-lactamase genes *bla*_*NDM*_ and *bla*_*VIM*_. A docking study revealed that pantoprazole could bind to and chelate zinc ions of New Delhi and Verona integron-encoded MBL (VIM) enzymes with higher affinity than the control drug captopril and with comparable affinity to the natural ligand meropenem, indicating the significant inhibitory activity of pantoprazole against metallo-β-lactamases. In conclusion, pantoprazole can be used in combination with meropenem as a new strategy for treating serious infections caused by metallo-β-lactamases producing *K. pneumoniae*.

## Introduction

*Klebsiella pneumoniae* is a facultative anaerobic Gram-negative bacterium of the family Enterobacteriaceae. It is non-motile and capsulated (Rapp and Urban, 2012). *K. pneumoniae* is recognized as a significant pathogen that is responsible for a range of clinical infections affecting various organ systems (Kang et al., 2006; Lila et al., 2023). Clinical manifestations often include healthcare-associated pneumonia, urinary tract infections, bloodstream infections, wound infections, meningitis, and intra-abdominal infections (Imai et al., 2019). Its ability to form a protective capsule, adhere to host tissues, and produce toxins contributes to its virulence (Victor et al., 2007). *K. pneumoniae* shows a significant increase in antibiotic resistance by different mechanisms, with resistance to β-lactamase as a major mechanism (Nordmann et al., 2009; Martin and Bachman, 2018). This leads to hindering effective treatment since *K*. *pneumoniae* infections are commonly treated by β-lactamase, including carbapenems and extended-spectrum cephalosporins (Bradford, 2001; Gasink et al., 2009). The emergence of multidrug-resistant strains poses a serious challenge, necessitating comprehensive innovative strategies for the development of effective treatment strategies (Khayat et al., 2022a; Alotaibi et al., 2023; Khayat et al., 2023; Nazeih et al., 2023).

Carbapenems are a class of broad-spectrum cell-wall antibiotics that belong to the β-lactam group, which also includes penicillins and cephalosporins. These antibiotics are structurally related to penicillins but possess a broader spectrum of activity against various bacteria, including both Gram-positive and Gram-negative pathogens (Breilh et al., 2013). The key features of carbapenems include their stability against many β-lactamases and their ability to penetrate bacterial cell walls effectively (Codjoe and Donkor, 2017). Carbapenems are considered “last-resort” antibiotics and are often reserved for serious infections caused by multidrug-resistant bacteria, particularly Gram-negative bacilli producing extended-spectrum β-lactamases (ESBLs), AmpC or carbapenemases (Zhanel et al., 2007). They are commonly used in hospital settings for conditions such as severe pneumonia, complicated intra-abdominal infections, complicated urinary tract infections, and septicemia (Hawkey and Livermore, 2012).

Despite their efficacy, the emergence of carbapenem-resistant bacteria predominantly among Gram-negative bacilli has been well documented (Codjoe and Donkor, 2017). Among the members of Enterobacteriaceae, *K. pneumoniae* is the most common carbapenem-resistant Enterobacterales (CRE). CRE was announced as an urgent global public health threat (Centers for Disease Control and Prevention, 2019; Rajab and Hegazy, 2023). Carbapenem resistance in *K. pneumoniae* is mediated by several mechanisms. The major mechanism is enzymatic hydrolysis by carbapenemases. Carbapenemases are grouped into A, D, and B classes. The Ambler class A includes *K*. *pneumoniae* carbapenemase (KPC), while class D includes the oxacillin enzyme (OXA) (Spagnolo et al., 2014; Logan and Weinstein, 2017). On the other hand, class B metallo-β-lactamases (MBLs) can degrade carbapenems in addition to most penicillins and cephalosporins. However, this class is stable to β-lactamase inhibitors, and they are inhibited by metal ion chelators (Queenan and Bush, 2007). MBLs include New Delhi metallo-β-lactamase (NDM-1) and Verona integron-encoded MBL (VIM) and acquire activity on imipenem (Poirel et al., 2000). Carbapenemase inhibitors are of much importance to restore the activity of carbapenems that are valuable in treating infections caused by *K*. *pneumoniae* because the classical β-lactamase inhibitors such as clavulanate, sulbactam, and tazobactam have no activity against *K*. *pneumoniae* carbapenemases (Drawz et al., 2010; Drawz et al., 2014).

Pantoprazole is a proton pump inhibitor (PPI). PPIs are drugs used to treat stomach acid-related disorders such as gastric ulcer, non-erosive reflux disease, as a prophylaxis against ulcers caused by non-steroidal anti-inflammatory drugs, and in combination with antibacterials for the therapy of *Helicobacter pylori* (Ward and Kearns, 2013; Strand et al., 2017). In addition to their classical uses, PPIs showed other activities against different types of pathogens. They were active against bacteria such as *Pseudomonas aeruginosa* and *Staphylococcus aureus* (Vidaillac et al., 2007; Sadiq et al., 2020), fungi such as *Candida albicans* and *Candida* spp. (Siavoshi et al., 2012), parasites such as *Entamoeba histolytica* (Pérez-Villanueva et al., 2011), and viruses such as SARS-CoV-2 (COVID-19) and rhinovirus (Sasaki et al., 2005; Aguila and Cua, 2020). This study aimed to investigate the ability of the PPI pantoprazole as a metallo-β-lactamase inhibitor compared to the well-known inhibitor captopril (García-Sáez et al., 2003; Klingler et al., 2015; Brem et al., 2016) to be used for treating CRE infections in combination with carbapenems.

## Materials and methods

### Bacterial isolates and chemicals

Two clinical isolates of *K*. *pneumoniae* sourced from the Microbiology and Immunology Department stock culture collection at the Faculty of Pharmacy, Zagazig University, were used. The clinical isolates were characterized using 16S rRNA gene sequencing, and the obtained results were deposited in GenBank (https://www.ncbi.nlm.nih.gov/) under accession numbers ON798797 and ON798801 (Abdel-Halim et al., 2022). Pantoprazole was supplied by Novartis, Egypt. Meropenem was supplied by AstraZeneca, Egypt. Captopril and dimethyl sulfoxide (DMSO) were purchased from Sigma Chemical Co. (St. Louis, MO, United States). Antibiotic disks and bacterial culture media were obtained from Oxoid, United Kingdom.

### Susceptibility test against different antimicrobial agents

The antimicrobial susceptibility testing of the test isolates was conducted for various classes of antimicrobial agents using the disk diffusion method, adhering to the guidelines set forth by the Clinical and Laboratory Standards Institute (CLSI) (Wayne, 2018). The used disks were meropenem (MEM, 10 μg), ceftriaxone (CRO, 30 μg), piperacillin–tazobactam (TZP, 100/10 μg), cefepime (FEP, 30 μg), cefoperazone (CFP, 75 μg), aztreonam (ATM, 30 μg), gentamicin (GN, 10 μg), amikacin (AK, 30 μg), trimethoprim–sulfamethoxazole (SXT, 1.25/23.75 μg), tetracycline (TE, 30 μg), tigecycline (TGC, 15 μg), levofloxacin (LEV, 5 μg), ofloxacin (OFX, 5 μg), chloramphenicol (C, 30 μg), and azithromycin (AZM, 15 μg).

### Determination of the minimum inhibitory concentration of meropenem against test isolates

The minimum inhibitory concentration (MIC) of meropenem was evaluated using the broth microdilution method according to CLSI guidelines (Thabit et al., 2022a; Cavalu et al., 2022). MICs were recognized as the lowest concentrations at which visible bacterial growth was inhibited.

### Effect of the sub-inhibitory concentration of pantoprazole on bacterial growth

To exclude the growth-inhibiting activity of pantoprazole or captopril, the effect of a sub-inhibitory concentration of pantoprazole (2 mg/mL) and captopril (1 mg/mL) on bacterial growth was evaluated by the turbidity measurement (Khayat et al., 2022b; Hegazy et al., 2022). In brief, the overnight cultures of the tested strains were diluted to obtain a turbidity equivalent to 0.5 McFarland standard, followed by 1/100 dilution in the Mueller–Hinton broth (MHB) containing pantoprazole or captopril and control MHB without the tested drugs. After incubation for 18 h at 37°C, turbidities were measured at 600 nm using a UV-vis microplate reader (Synergy HT, BioTek) and compared.

### Combined disk test

To assess the potential synergy between each captopril or pantoprazole and meropenem, the combined disk test was performed (Boonyanugomol et al., 2017). MH agar plates were prepared containing a final concentration of 2 mg/mL pantoprazole or a final concentration of 1 mg/mL captopril, and control plates without pantoprazole were prepared. Adjusted bacterial suspensions (0.5 McFarland standard) were prepared, and 100 μL aliquots were delivered and spread on the surface of the plates. A disk containing 10 mg of meropenem was placed on the center of the test and control plates. After 18 h of incubation at 37°C, the diameters of the inhibition zone were measured and photographed.

### Quantitative carbapenemase inhibition assay in the crude periplasmic extract

Crude periplasmic extracts of isolates were prepared (Bernabeu et al., 2012). A loopful of the test isolates was inoculated in 10 mL of MHB that were incubated with shaking at 37°C for 18 h. The suspensions were centrifuged to harvest the pellets that were resuspended in 0.5 mL of phosphate buffer (100 mM, pH 7.0) combined with 50 µM ZnSO_4_ in a microcentrifuge tube and sonicated (ultrasonic system UP100H, Hielscher Ultrasonic Technology, Teltow, Germany) at 40 W for 1.5 min, with a pulse of 0.5 s. After centrifugation, the supernatants were used to assess the meropenem hydrolysis activity in the absence or presence of pantoprazole (2 mg/mL) or captopril (1 mg/mL) at 297 nm using the UV-vis spectrophotometer (Synergy HT, BioTek) (Denny et al., 2002). Then, 100 µl aliquots were transferred to a 96-well microtitre plate, and the plates were incubated with pantoprazole (2 mg/mL) in 0.5% DMSO at 37°C for 30 min. Meropenem was added (500 μg/mL), and the solutions were incubated at 37°C for 1 h. The absorbances of solutions containing pantoprazole or captopril (test) and 0.5% DMSO (vehicle control) were detected at 297 nm. The percentage of meropenem hydrolysis inhibition was calculated using the following formula:

$$\%\text{ of inhibition}=\left[\left(O.D.\text{of treatment }-\;O.D.\text{vehicle control}\right)/O.D.\text{of treatment}\right]\times 100.$$

### Effect of pantoprazole on the susceptibility of bacteria to meropenem

The influence of the sub-MICs of pantoprazole or captopril on the MIC of meropenem against the tested bacterial isolates was evaluated by using the broth microdilution method as previously described (Khayyat et al., 2021; Elfaky et al., 2022).

### Quantitative RT-PCR of metallo-β-lactamase genes

To analyze the relative expression levels of carbapenemase genes *bla*_*NDM*_ and *bla*_*VIM*_ in the *K. pneumoniae* ON798797 strain in the absence or presence of 1 mg/mL of pantoprazole, quantitative real-time-PCR (qRT-PCR) was employed. The primers for the two metallo-β-lactamase genes were obtained from Integrated DNA Technologies (IDT) (Coralville, Iowa, United States). The sequences of the primers are listed in Table 1 (Amira et al., 2016; Abbas et al., 2019). The housekeeping gene *rpoB* was used to normalize the relative expression levels of the tested genes. The StepOne Real-Time PCR System (Applied Biosystems, United States) was utilized, and the protocol of the SensiFAST™ SYBR^®^ Hi-ROX One-Step Kit (Bioline, United Kingdom) was followed (Askoura et al., 2022; Elfaky et al., 2023). The thermocycling conditions were as follows: 3 min at 95 °C for enzyme activation, followed by 40 cycles (denaturation for 15  s at 95 °C, annealing for 30 s at 50–60 C, and extension for 30  s at 72 °C). The relative gene expression was determined using the comparative threshold cycle method, as outlined in the reference (Livak and Schmittgen, 2001; Hatipoglu et al., 2015; Thabit et al., 2022b).

**TABLE 1:** Primers used in the genotypic detection of carbapenemase genes.

Primer	Sequence (5′–3′)
*bla*_NDM_ F	GCA​CAC​TTC​CTA​TCT​CGA​CAT​GC
*bla*_NDM_ R	CCA​TAC​CGC​CCA​TCT​TGT​CC
*bla*_VIM_ F	GAT​GGT​GTT​TGG​TCG​CAT​A
*bla*_VIM_ R	CGAATGCGCAGCACCAG
*rpoB F*	AAC​CCG​CTG​TCT​GAG​ATT​AC
*rpoB R*	GGC​GTT​TCG​ATC​GGA​CAT​A

### *In silico* study

The crystal structure of hydrolase enzymes, i.e., *K. pneumoniae* apo NDM-1 (Protein Data Bank (PDB) ID: 3SPU) at a resolution of 2.10 Å and VIM-2 (PDB ID: 5YD7) at a resolution of 1.70 Å, was retrieved from the PDB (https://www.rcsb.org/) in PDB format. Each of the pantoprazole, captopril, and meropenem structures were drawn using MarvinSketch of Marvin Suite (http://www.chemaxon.com) to generate a three-dimensional (3D) conformer for each with the lowest energy and then saved in Mol2 format (King and Strynadka, 2011; Chen et al., 2020; Singh et al., 2022).

After the removal of all water molecules, the crystal 3D structure of the New Delhi metallo-β-lactamase enzyme underwent protonation with their standard geometry, followed by energy minimization. The dock module of Molecular Operating Environment (MOE) version MOE 2019.0102 was utilized (Inc, 2016). The rigid binding pocket of the protein was accommodated by the three tested compounds through the flexible ligand mode. Poses were generated during the placement phase based on ligand conformations. The force field-based scoring function GBVI/WSA ΔG was employed to estimate the free energy of binding for the ligand from a specific analysis (Labute, 2008; Mahomoodally et al., 2018; Bender et al., 2023).

### Statistical analysis

All the experimental investigations were conducted in triplicates, and the mean and standard errors were calculated. One-way ANOVA statistical test, unless otherwise mentioned, was employed to attest the significance, where *p* < 0.05 was considered significant.

## Results

### Susceptibility to antibiotics

The disk diffusion method was carried out to investigate the one susceptibility profile of the test isolates (Table 2) to various antibiotics. Both the two tested *K. pneumoniae* isolates were multi-drug resistant (MDR), showing resistance to meropenem.

**TABLE 2:** Antibiotic susceptibility profile of the clinical isolates of *Klebsiella pneumoniae.*

Isolate code	Anti-microbial agent														
	MEM	CFP	CRO	TZP	FEP	ATM	OFX	LEV	AK	CN	TGC	TE	SXT	AZM	C
**ON798797**	R	R	R	R	R	R	R	R	S	S	I	R	R	R	S
**ON798801**	R	R	R	R	R	R	R	R	R	R	I	I	R	R	R

MEM, meropenem; CRO, ceftriaxone; TZP, piperacillin–tazobactam; FEP, cefepime; CFP, cefoperazone; ATM, aztreonam; GN, gentamicin; AK, amikacin; SXT, trimethoprim–sulfamethoxazole; TE, tetracycline; TGC, tigecycline; LEV, levofloxacin; OFX, ofloxacin; C, chloramphenicol; AZM, azithromycin.

### Determination of the MICs of meropenem and pantoprazole against test isolates

The MICs of meropenem and the test drug pantoprazole against the test isolates were determined according to CLSI guidelines, and the results are shown in Table 3.

**TABLE 3:** Minimum inhibitory concentrations of meropenem and pantoprazole against tested *Klebsiella pneumoniae* isolates.

Isolate code	Minimum inhibitory concentration (MIC)		
	Meropenem	Pantoprazole	Captopril
**ON798797**	256 μg/mL	16 mg/mL	8 mg/mL
**ON798801**	128 μg/mL	16 mg/mL	8 mg/mL

### The effect of pantoprazole and captopril at a sub-MIC on bacterial growth and viability

**FIGURE 1:**
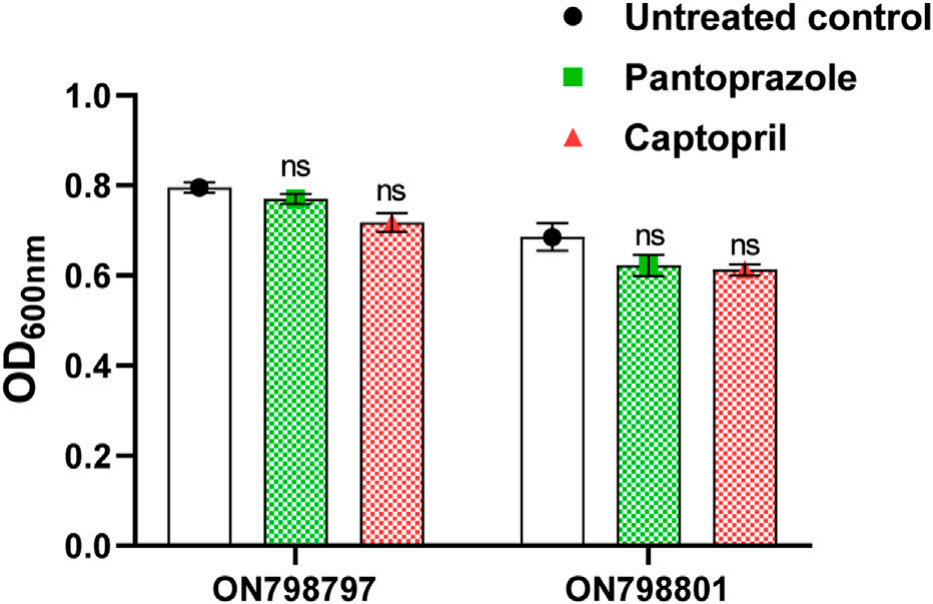
Effect of the sub-minimum inhibitory concentration (MIC) of pantoprazole and captopril on the growth of tested isolates. No significant difference was found in the growth of the test isolate by either captopril or pantoprazole; non-significant (ns): *p* > 0.05.

To exclude the possible effect of captopril and pantoprazole on metallo-β-lactamases due to growth inhibition, the effect of the drugs on viability was investigated by measuring the turbidities of suspensions in the presence and absence of sub-minimum inhibitory concentrations (1/8 MICs) of the tested drugs (2 mg/mL pantoprazole and 1 mg/mL captopril). There were no significant differences between OD_600_ in control and test samples. This indicates the lack of the effect of captopril and pantoprazole on the bacterial growth (Figure 1).

### Combined disk test

**FIGURE 2:**
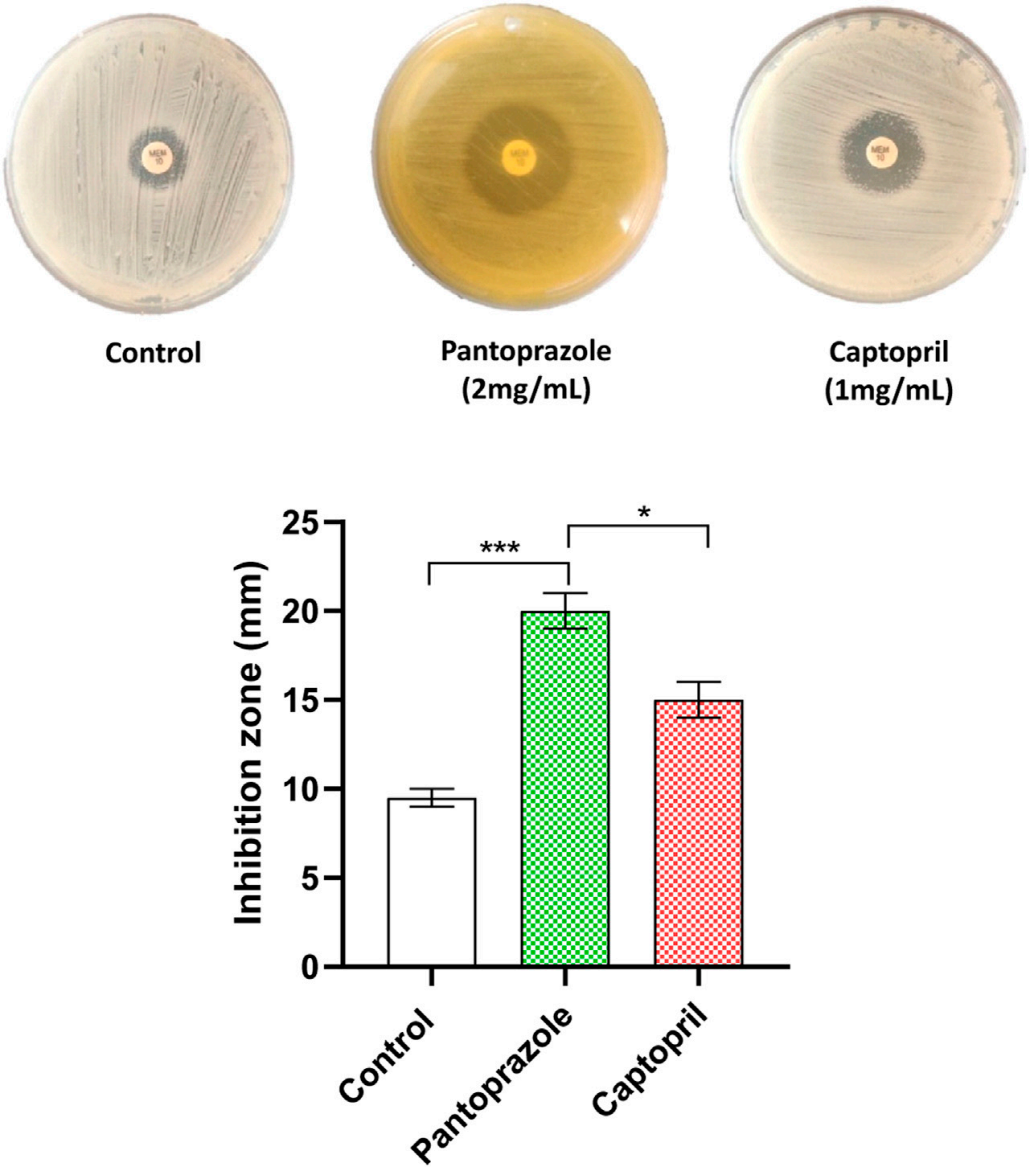
Potentiation of meropenem antibacterial activity against the tested isolates by the combined disk test. A significant increase in the inhibition zone diameter of meropenem was observed in plates with the tested drugs compared to control plates. *: *p* < 0.05 and ***: *p* < 0.001.

The possible synergy between meropenem and the tested drugs was tested by the combined disk test. The inhibition zone produced by meropenem was significantly increased from a mean diameter of 10 mm in the control plates to mean diameters of 20 mm for pantoprazole and 16 mm for captopril at 1/8 MICs (Figure 2). Importantly, pantoprazole significantly potentiates the meropenem activity compared to captopril.

### Pantoprazole inhibited the hydrolytic activity of metallo-β-lactamases in the crude periplasmic extract of test isolates

**FIGURE 3:**
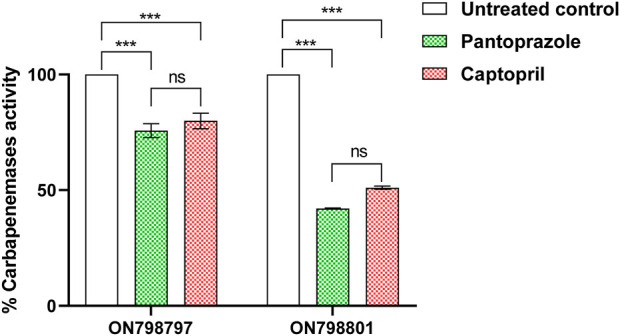
Inhibition of carbapenemase by pantoprazole and captopril. The sub-minimum inhibitory concentration of pantoprazole showed higher inhibiting activities than that of captopril. ***: *p* < 0.001.

The effect of both pantoprazole (2 mg/mL) and captopril (1 mg/mL) was assessed, showing that they significantly inhibited meropenem hydrolysis by carbapenemase-mediated hydrolysis of meropenem in the crude periplasmic extract of tested bacterial isolates. Pantoprazole was more active as an inhibitor of carbapenemase, showing 25% and 60% inhibition compared to captopril, which showed inhibition percentages of 20% and 50% in tested isolates ON798801 and ON798797, respectively (Figure 3).

### The synergy between meropenem and tested drugs

To investigate the potential potentiation of meropenem by tested drugs, the MIC of meropenem was determined in the presence of the tested drugs. Pantoprazole at 1/8 MIC decreased the MIC of meropenem by 4-fold, a lower potentiating effect than that of captopril at 1/8 MIC (8-fold), as shown in Table 4.

**TABLE 4:** Combined effect of meropenem with tested drugs on the susceptibility of *Klebsiella pneumoniae* isolates.

Isolate code	Minimum inhibitory concentration (MIC) (µg/mL)		
	Meropenem	Meropenem + pantoprazole (2 mg/mL)	Meropenem + captopril (1 mg/mL)
**ON798797**	256	64	32
**ON798801**	128	32	16

### Pantoprazole downregulated metallo-β-lactamase genes *bla*_*VIM*_ and *bla*_*NDM*_

**FIGURE 4:**
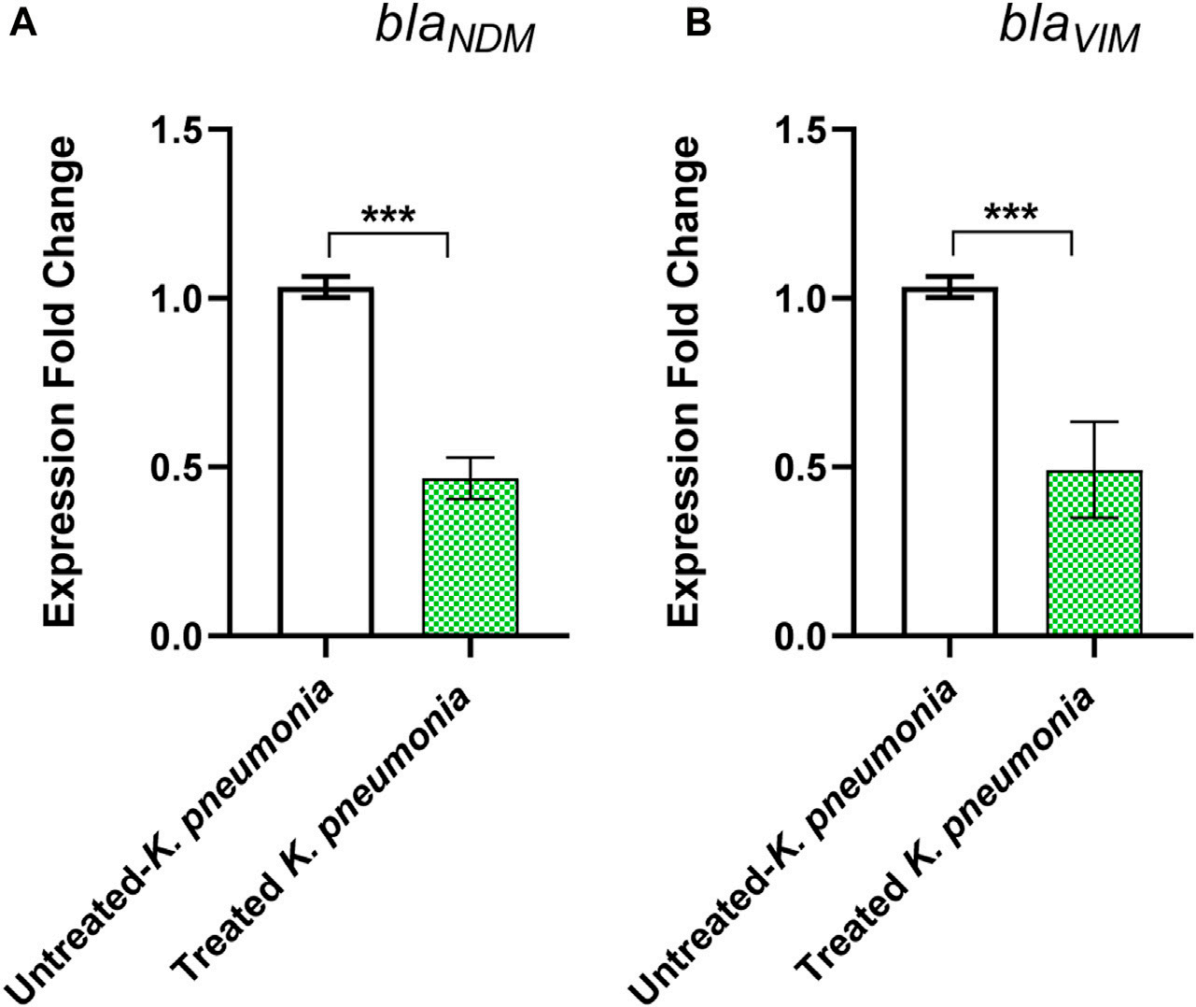
Downregulation of **(A)** *bla*_*NDM*_ and **(B)** *bla*_*VIM*_ genes by pantoprazole. Pantoprazole downregulated the expression of both genes. ***: *p* < 0.001.

To further confirm the metallo-β-lactamase inhibiting activity of pantoprazole at the molecular level, the relative expression of the genes *bla*_*VIM*_ and *bla*_*NDM*_ in the strain ON798797 was estimated in the presence and absence of pantoprazole (1 mg/mL) by quantitative real-time PCR. It was found that pantoprazole downregulated the expression of both *bla*_*VIM*_ and *bla*_*NDM*_ by approximately 2-fold (Figure 4).

### Pantoprazole chelation of zinc ions in New Delhi metallo-β-lactamase and VIM enzymes in the *in silico* study

Molecular modeling simulation is traditionally carried out to explore the interactions of ligands with their respective binding sites in the crystal structures of the enzymes (Mansour et al., 2023) Figure 5 (upper panel) shows that the docking results of pantoprazole against the crystal structure of the New Delhi metallo β-lactamase receptor displayed a unique type of halogen bonds (El-Abd et al., 2022) between the backbone of the conserved amino acid Asn220 and one of the fluorine atoms in the terminal difluoromethoxy moiety at position 5 of the benzimidazole scaffold. In addition, an arene–H-bond constructed between the non-classical Lewis base pyridine ring and the conserved amino acid Ala215 and the conspicuous hydrophobic/hydrophilic interactions expressed by cyan-shaded amino acids from the receptor side and the blue-shaded moieties from the ligand side improved the overall recognition and enhanced the ligand/receptor complex stability to score free-binding energy of −10.3401031 kcal/mol.

**FIGURE 5:**
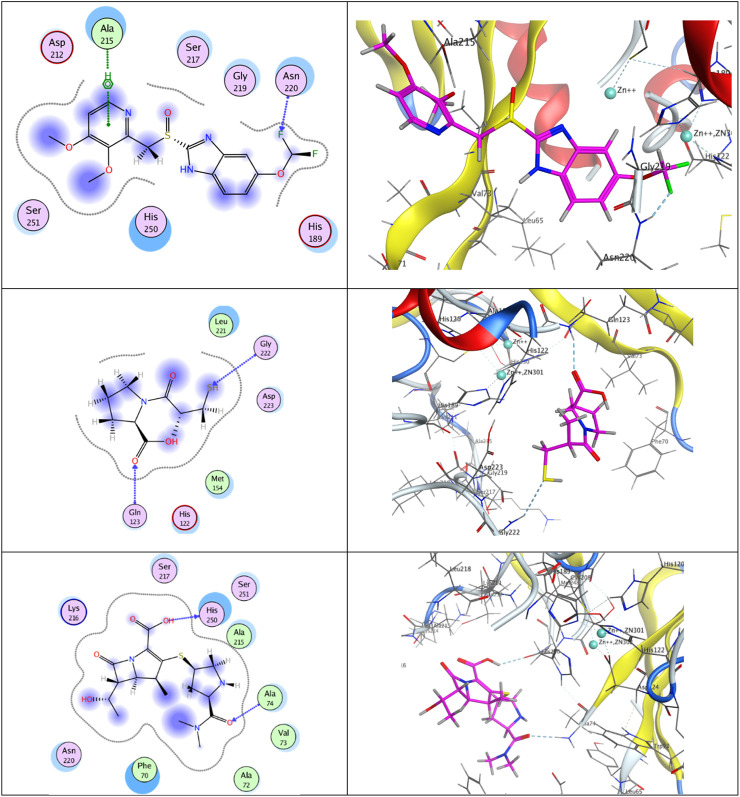
Putative binding modes of compounds pantoprazole (upper panel), captopril (middle panel), and meropenem (lower panel) with the receptor pocket of apo New Delhi metallo-β-lactamase-1 (NDM-1) crystal structure (Protein Data Bank (PDB) ID: 3SPU).

Concerning captopril (middle panel), two H-bonds were built between the mercapto group of the H-bond acceptor and the *sp* ^*2*^-hybridized oxygen atom of the carboxylic group with the backbones of the conserved H-bond donor amino acids Gly222 and Gln123, respectively, giving rise to a total binding energy of −8.00626659 kcal/mol.

Analogously, meropenem (lower panel) exhibited two H-bonds between the *sp* ^*2*^-hybridized oxygen atom in the dimethyl carbamoyl moiety of the H-bond acceptor and the H-bond donor OH group in the carboxylic moiety at position 2 and the backbones of the conserved amino acids Ala74 and His250, respectively, ending up with a total score of free-binding energy of −10.7858448 kcal/mol.

It is noteworthy that although the three ligands are rich in H-bond acceptors and donor sites, meropenem and pantoprazole have achieved higher binding activities than captopril. This may be attributed to the steric effect, the bulkiness of the moieties, and the appropriate spacers of the two privileged ligands, meropenem and pantoprazole, that steered them to well-fit positioning inside the active site.

**FIGURE 6:**
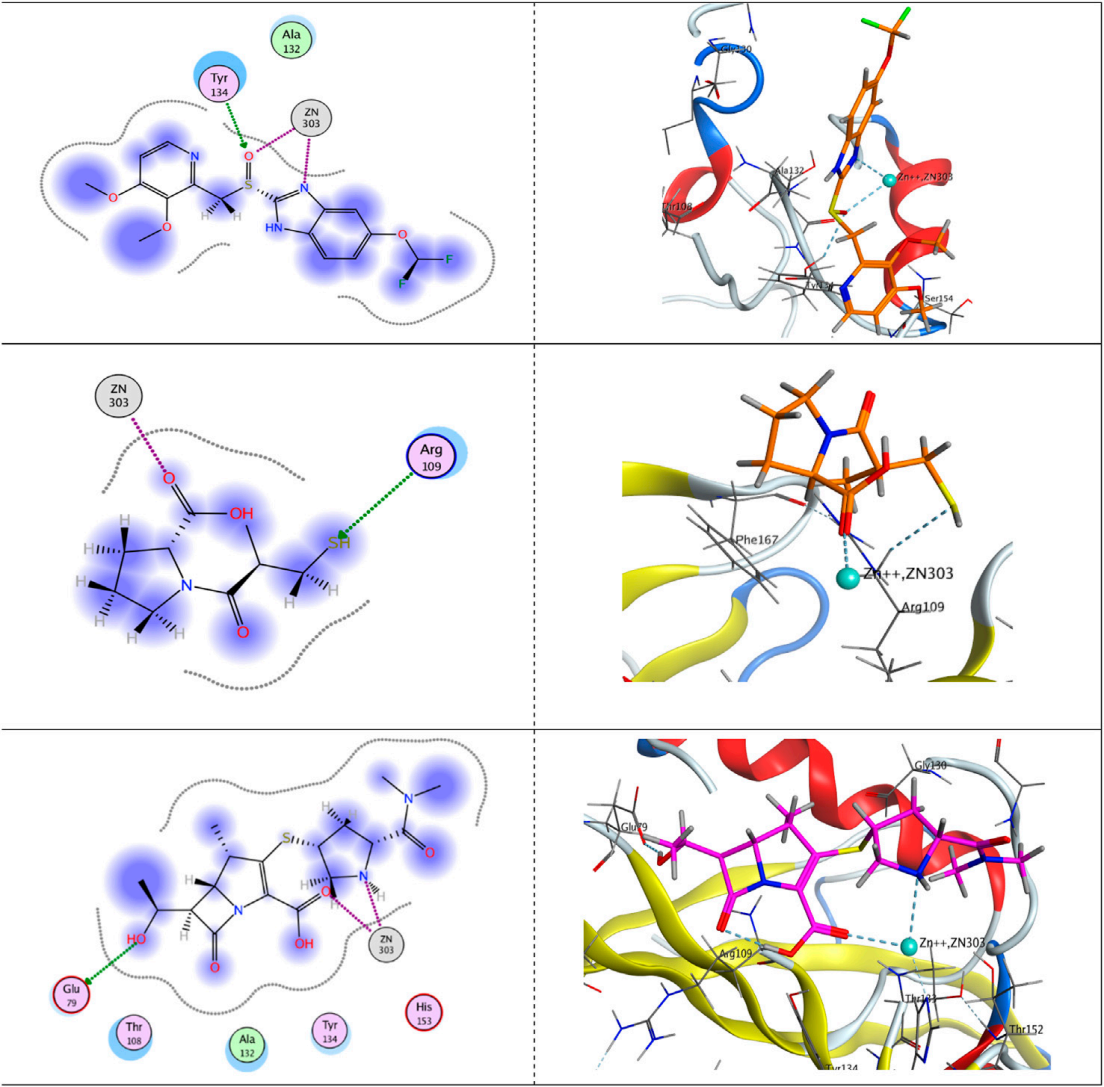
Putative binding modes of pantoprazole (upper panel), captopril (middle panel), and meropenem (lower panel) with the receptor pocket of the Verona integron-encoded MBL-2 (VIM-2) crystal structure (PDB ID: 5YD7).

On the other hand, upon docking the three ligands against the crystal structure of the hydrolase enzyme VIM-2 (PDB ID: 5YD7), as shown in Figure 6, we found that pantoprazole appeared as a bidentate ligand, with two bonds with Zn^++^ constructed *via* its chelating centers; the *sp* ^*2*^-hybridized oxygen atom of the sulfinyl moiety and *sp* ^*2*^-hybridized nitrogen atom of the imidazole ring. Moreover, the first chelating center formed another H-bond with the side chain of the H-bond donor-conserved amino acid Tyr134, ending up with a total free-binding energy of −7.80613756 kcal/mol.

Concerning captopril, it was featured as a monodentate ligand and formed one chelating bond with Zn^++^ with its *sp* ^*2*^-hybridized oxygen atom of the carboxylic group. However, the active mercapto group formed acted as an H-bond acceptor and formed an H-bond with the side chain of the conserved amino acid Arg109, giving rise to a total free-binding energy of −7.91857481 kcal/mol.

Eventually, Zn^++^ formed two chelating bonds with the ligand meropenem, the first with *the sp* ^*2*^-hybridized oxygen atom of the carboxylic group and the latter with the *sp* ^*3*^-hybridized nitrogen atom of the pyrrolidine ring. Meropenem is represented as a bidentate ligand. Furthermore, an H-bond was displayed between the terminal OH group and the side chain of the H-bond acceptor amino acid Glu79, augmenting a total free-binding energy of up to −8.91452599 kcal/mol.

## Discussion

The widespread dissemination of carbapenemase-producing *K. pneumoniae* within the Enterobacteriaceae family undermines the effectiveness of carbapenem therapy (Rossolini, 2005). Moreover, the alternative therapeutic options for serious infections caused by this bacterium are so limited because they are insensitive to almost all other classes of antimicrobial agents (Cornaglia et al., 2007; Almalki et al., 2022; Cavalu et al., 2022). This presents a significant public health concern due to the high mortality rates associated with these infections. Currently, only a few antibiotics, such as tigecycline and polymyxins, are utilized to combat carbapenem-resistant *K. pneumoniae*. However, tigecycline, a broad-spectrum tetracycline derivative, faces challenges in achieving adequate concentrations in blood, respiratory, and urinary tracts, rendering it unsuitable for treating infections such as pneumonia, bacteremia, and urinary tract infections (Barbour et al., 2009; Freire et al., 2010). Polymyxins are cationic cyclic polypeptides of which polymyxin B and colistin are used for the therapy against Gram-negative infections (Akajagbor et al., 2013). However, colistin causes nephrotoxicity and neurotoxicity that limit its use (Garonzik et al., 2011).

In light of the serious side effects and limitations of tigecycline and polymyxins, it is vital to search for other alternative therapeutic options. Two combinations are approved for treating carbapenemase-producing bacteria, and the first is meropenem/vaborbactam for complicated urinary tract infections in addition to pneumonia (Vázquez-Ucha et al., 2020). However, meropenem/vaborbactam is inactive against class-B or D carbapenemases (Lomovskaya et al., 2017). The second combination is relebactam with imipenem. Relebactam could not inhibit class-D OXA-48 β-lactamases; however, its inhibiting activities against class-A and class-C β-lactamases are pronounced (Papp-Wallace et al., 2018).

In this study, two clinical *K*. *pneumoniae* isolates were found to be multidrug-resistant and meropenem-resistant. Two drugs were used, and the well-reported metallo-β-lactamase inhibitor captopril and the proton pump inhibitor pantoprazole were considered a candidate for metallo-β-lactamase inhibition. They were used at sub-inhibitory concentrations that did not affect cell growth to guarantee that any possible effect on metallo-β-lactamases is not due to the interference with cell growth. Both pantoprazole and captopril synergized meropenem when combined with it in the combined disk method, and they reduced its MIC. When investigated for their activities against the inhibition of hydrolysis of carbapenem meropenem by the carbapenemase enzyme, pantoprazole showed a higher ability than captopril to protect meropenem from hydrolysis mediated by carbapenemase. To further confirm the action of pantoprazole on metallo-β lactamases, quantitative real-time PCR was performed to investigate if pantoprazole can downregulate the genes *bla*_*NDM*_ and *bla*_*VIM*_ that encode for the metallo-β-lactamase enzymes. Interestingly, pantoprazole could decrease the expression of the tested genes to a significant level. The observed decrease in MBL gene expression following treatment with pantoprazole substantiates its inhibitory effect on MBL at the molecular level. This indicates that its activity is not attributed to chemical interactions or other factors but rather to the direct interaction with the genes responsible for encoding MBL enzymes. Consequently, this suggests that pantoprazole is a suitable candidate for therapeutic intervention against MBL-producing *K*. *pneumoniae* infections.

The proposed mechanism of action of pantoprazole against metallo-β-lactamases is its ability to chelate metals. Metallo-β-lactamases have zinc ions in their active sites, which is essential for activity. As a result, the chelation of zinc can inhibit the action of metallo-β-lactamases. This was well documented in previous studies. Captopril was used in this study as a standard metallo-β-lactamase inhibitor. Captopril was previously found to inhibit metallo-β-lactamases NDM-1, VIM-1, and IMP-7 through zinc chelation by merit of its free thiol group (García-Sáez et al., 2003; Klingler et al., 2015; Brem et al., 2016). Furthermore, the drug tiopronin, a medication used in the prevention of renal stones and the treatment of heavy-metal poisoning, is a good inhibitor of the metallo-β-lactamases NDM-1, VIM-1, and IMP-7 (Brem et al., 2016). Pantoprazole is a benzimidazole compound. Benzimidazole compounds were previously found to chelate metal ions (Sánchez-Moreno et al., 2004; Chkirate and Essassi, 2022). This was further confirmed by an *in silico* study that proved the possible strong binding of pantoprazole with zinc ions in the active site of the New Delhi metallo-β-lactamase enzyme. Pantoprazole showed binding affinity more or less similar to that of New Delhi metallo-β-lactamase to the natural ligand meropenem, reflecting the possibility of pantoprazole to act as a potent inhibitor of metallo-β-lactamases. This binding activity is higher than that of the previously reported metallo-β-lactamase inhibitor captopril. Moreover, pantoprazole showed a more potent chelation of zinc ions in the VIM-2 enzyme than did captopril. To summarize the results of the *in silico* study, successful chelating centers were found. These centers are the *sp* ^*2*^-hybridized oxygen atom of the sulfinyl moiety and *sp* ^*2*^-hybridized nitrogen atom of the imidazole ring in pantoprazole, the *sp* ^*2*^-hybridized oxygen atom of the carboxylic group in captopril in addition to the *sp* ^*2*^-hybridized oxygen atom of the carboxylic group, and the *sp* ^*3*^-hybridized nitrogen atom of the pyrrolidine ring in meropenem. As chelation with Zn^++^ leads to the formation of a water-soluble complex, chelation not only impedes the catalytic role for this heavy metal to this hydrolase enzyme but also enhances the excretion of Zn^++^, and this is the mainstay of chelation therapy (Flora and Pachauri, 2010). The overall conclusion from the docking study is that pantoprazole is a promising inhibitor of metallo-β-lactamases in terms of bulkiness, steric effect, spacers, binding mode and affinity, and the number of chelating centers. This activity is more pronounced than that of captopril.

In conclusion, pantoprazole can be used in combination with meropenem to treat serious resistant *K. pneumoniae* infections due to its ability to inhibit metallo-β-lactamases. However, the impact of pantoprazole was not investigated in conjunction with other carbapenems. It is reasonable to anticipate its potential effectiveness when used in combination with them. Further pharmacological studies are needed to prove its efficacy and safety before clinical application.

## Data Availability

The original contributions presented in the study are included in the article/Supplementary Material; further inquiries can be directed to the corresponding author.
